# Separation of Berberine Hydrochloride and Tetrahydropalmatine and Their Quantitative Analysis with Thin Layer Chromatography Involved with Ionic Liquids

**DOI:** 10.1155/2015/642401

**Published:** 2015-11-01

**Authors:** Jing Lu, Hong-yan Ma, Wei Zhang, Zhi-guo Ma, Shun Yao

**Affiliations:** ^1^School of Chemical Engineering, Sichuan University, Chengdu 610065, China; ^2^School of Traditional Chinese Medicine, Guangdong Pharmaceutical University, Guangzhou 510006, China; ^3^College of Pharmacy, Jinan University, Guangzhou 510632, China

## Abstract

[BMIM]OH was used in mobile and stationary phase of thin layer chromatography (TLC) to analyze berberine hydrochloride and tetrahydropalmatine for the first time. Supported imidazole ionic liquid with hydroxide ion on silica gel (SiO_2_·Im^+^·OH^−^) was synthesized through simple procedure and characterized by Fourier transform infrared spectroscopy (FT-IR), elemental analysis, and scanning electron microscope (SEM). Moreover, on the plates prepared by SiO_2_·Im^+^·OH^−^, the contents of the above alkaloids in the Chinese patent medicine (CPM) of “Stomacheasy” capsule were successfully determined by TLC scanner. The key conditions and chromatographic behaviors were also investigated in detail. According to similar ways, ionic liquids (ILs) also can be used in other planar chromatographies in two modes. This study is expected to be helpful in expanding the application of IL and its bonded silica gel in TLC separation field.

## 1. Introduction

Berberine hydrochloride and tetrahydropalmatine are two natural bioactive nitrogen-containing compounds belonging to the protoberberine hydrochloride group of isoquinoline alkaloids, and they are found in many plants which have traditional uses in Chinese herbal medicine (e.g., Rhizoma Coptidis and Rhizoma Corydalis). The main structural difference between them is that the former is a quaternary ammonium salt form. Berberine hydrochloride has shown obvious biological activity against fungal infections,* Candida albicans*, yeast, parasites, and bacterial/viral infections. Tetrahydropalmatine has been demonstrated to possess analgesic effects and be beneficial in the treatment of heart disease and liver damage. Some Chinese patent medicines also contain the two isoquinoline alkaloids, which are usually selected as the major symbolic components in quality control. The determination approaches about them on TLC have been widely investigated and applied for a very long time. However, the new method and chromatographic medium are still looked forward to by researchers and analysts, which can provide the new driving force for drug analysis (especially for TCL as a relatively old technique). Although silica gel is popularly used for TLC analysis as stationary, surface silanol groups are nonbeneficial for the perfect separation of polar and basic compounds, which could form strong absorption and unavoidable tailing. Fortunately, surface functionalisation of Si-OH groups has given rise to a new generation of sorbents for planar chromatographic applications. More new functional silica gels need to be developed for various application requirements in modern TLC.

Though TLC is an important qualitative and quantitative technique in pharmacopoeia, the research about new TLC separation methods is relatively less in recent years. On the other hand, the reports about ionic liquids (ILs) are increasing more and more. They have been taken a step forward and been immobilized on solid supports, thereby providing new kinds of materials with interesting properties. These new materials or agents have been applied as stationary phase or mobile phase in various chromatographic analyses [1–3] and related pretreatment [4–6]. The supported ionic liquids (SILs) are prepared by immobilizing ionic liquid moieties onto solid supports via covalent bonds, which have been successfully used in separation work due to their characteristic cations and anions [7–9]. Imidazolium series is the most studied cation for the modification of separation materials, but there is no report about the TLC application of related ILs in the analysis of natural isoquinoline alkaloids up to now.

In view of above status, alkylimidazole ILs were used as mobile and stationary phase of TLC to analyze berberine hydrochloride, tetrahydropalmatine, and related Chinese patent medicine for the first time. [BMIM]OH was immobilized on conventional TLC silica gel and characterized by Fourier transform infrared spectroscopy (FT-IR), elemental analysis, and scanning electron microscope (SEM). Moreover, the key problems and performance of ILs were explored and discussed in detail. The research is expected to be helpful in extending the application of ILs in the daily analytical work for natural medicine.

## 2. Experimental

### 2.1. Materials and Apparatus

N-Methylimidazole (98%) was purchased from Hanhong Chemical Technology Co., Ltd. (Shanghai, China). 3-Chloropropyltrimethoxysilane, potassium hexafluorophosphate (KPF_6_), and sodium tetrafluoroborate (NaBF_4_) were purchased from Sigma-Aldrich. Commercial silica gel G TLC plates and amorphous TLC silica gel were obtained from Haiyang Chemical Reagent Factory (Qingdao, China) with a surface area of 300~400 m^2^/g. All the other chemicals (AR grade) were commercially available and used without further purification. Experimental water was purified before use with Millipore (Bedford, MA) ultrapure water system (0.4 mm filter).

Element analysis of C, H, N, and S was determined by EA3000 elemental analyzer (EuroVector S.p.A., USA). Scanning electron microscopy (SEM) photograph of silica particles was captured with JSM-7500F Field Emission Microscope (JEOL, Japan). Thermogravimetric analysis (TGA) was performed on a TG 209 Fl Iris (NETZSCH Company, Germany) with a heating rate of 10°C/min from 30 to 800°C under N_2_. FT-IR spectra were obtained with NEXUS 670 spectrometer (Nicolet Instrument Corporation, Fitchburg, USA) using potassium bromide pellets. The quantitative analysis of the two alkaloids was operated on CS-9301 dual-wavelength flying spot TLC scanner (Shimadzu, Japan). SPSS 17.0 software was used to perform analysis of variance (ANOVA) for the quantitative results.

### 2.2. Synthesis of IL and SILs

[BMIM]OH, [BMIM]BF_4_, [BMIM]Br, and [BMIM]PF_6_ were synthesized according to the reported procedures [10–15]. All ILs were dried for 4 h under vacuum at 90°C and stored in closed desiccators before use. The purity of these ILs was determined by HPLC and their purity was greater than 98.0% (w/w). These commercial ILs also can be easily purchased from chemical reagent companies.

The silica gel was firstly activated by the following procedure: 10.0 g TLC silica was stirred with 250 mL nitric acid-water (50 : 50, V/V) at room temperature for 2 h and refluxed for 8 h. Then, the activated silica was collected by filtration and washed thoroughly with distilled water and acetone successively. Finally, the activated silica was dried under vacuum overnight prior to use. The aim of pretreatment is to enhance the content of silanol groups on the silica surface and to eliminate metal oxide and nitrogenous impurity.

[BMIM]OH based SIL (SiO_2_·Im^+^·OH^−^) was prepared as follows: equal molar amounts of N-methylimidazole and 3-chloropropyltrimethoxysilane were stirred at 100°C under N_2_ atmosphere. After the complete reaction, the residuals were washed by ethyl acetate for three times and evaporated under vacuum to obtain viscous pale yellow liquid Im^+^·Cl^−^. Then, Im^+^·Cl^−^ was added to the silica gel suspension in anhydrous toluene and this mixture was refluxed for 24 h. After filtering, the resulting materials were washed with toluene and acetone and then dried under vacuum at 60°C to obtain SiO_2_·Im^+^·Cl^−^. Then, SiO_2_·Im^+^·Cl^−^ was anion-exchanged by stirring it with KOH-acetonitrile solution; the suspension was filtered and washed with water and acetone. Finally, the particles were dried under vacuum at room temperature for 12 h to afford SiO_2_·Im^+^·OH^−^.

### 2.3. Preparation of TLC with SIL

Dry SiO_2_·Im^+^·OH^−^ particles were firstly sieved to 300~400 mesh before use. Then, TLC glass plates (width: 5 cm or 10 cm; length: 10 cm or 15 cm) were precoated with the SIL of 0.25 mm thickness by automatic spreader as follows: SIL particles and 3 parts of 0.3% carboxymethyl cellulose sodium salt (CMC-Na) solution were mixed uniformly by shaking vigorously for 30 seconds in a glass-stoppered conical flask, and then the slurry was transferred to the spreader. The precoated plates were placed in the shade until dryness and stored in a desiccator for use.

### 2.4. Preparation of Standard Solution

The standard compounds for TLC determinations were berberine hydrochloride and tetrahydropalmatine (both purities >98.5%), which were supplied by Mansite Pharmaceutical Co., Ltd. (Chengdu, China), and accurately weighed and dissolved in methanol as the standard stock solutions. Assessed by spectrophotometric assay, the stock solutions were stable for at least 15 days when stored in the dark at −5°C. Working solutions of berberine hydrochloride and tetrahydropalmatine were prepared by diluting the standard stock solution to 0.1, 0.5, 1, 2, 4, and 8 mg/mL.

### 2.5. Traditional TLC Analysis for the Two Alkaloids

2 *μ*L of 1 mg/mL two standard solutions was spotted on the silica gel G TLC plates with glass spotting pin. After the saturation of the covered developing chamber with solvent vapors for 30 min, the spots began to be developed with* n*-butanol-acetic acid-water (7 : 1 : 2, V/V, for berberine hydrochloride) or* n*-hexane-chloroform-methanol (10 : 6 : 1, V/V, for tetrahydropalmatine), respectively, until the solvent was about half a centimeter below the top of the plates. Then, the plates were taken out from the chamber and dried in the air. The spots of the same compounds in the analyzed samples would appear in the horizontal position with the same *R*_*f*_ value of the corresponding spots of standard compounds. In some conditions, the spots could be colored with Dragendorff's reagent or iodine vapor before the quantitation procedure.

### 2.6. Extraction of Target Alkaloids for Chinese Patent Medicine

“Stomacheasy” capsule is purchased from local drug store and made from the herbal materials mainly including* Cortex Phellodendri* Chinensis (which mainly contains berberine hydrochloride) and Rhizoma Corydalis (which mainly contains tetrahydropalmatine), which is used to treat atrophic gastritis, inappetence, hiccup singultation, abdominal distension, and pain as a common Chinese patent drug. 1 g contents of “Stomacheasy” capsule were first weighed accurately and placed in conical flask with cover. Then, 50 mL of methanol was added in the flask and weighed, and the contents were extracted with it for 30 min in ultrasound wave (250 W, 50 KHz). After the system was cooled to room temperature, the flask was weighed again and loss of methanol was compensated. The extract was filtered and 1 mL filtrate was transferred to a 10 mL volumetric flask, then diluted with methanol to volume, and stored for further analysis.

## 3. Results and Discussion

### 3.1. Separation Performance of IL as Mobile Phase

**Figure 1:**
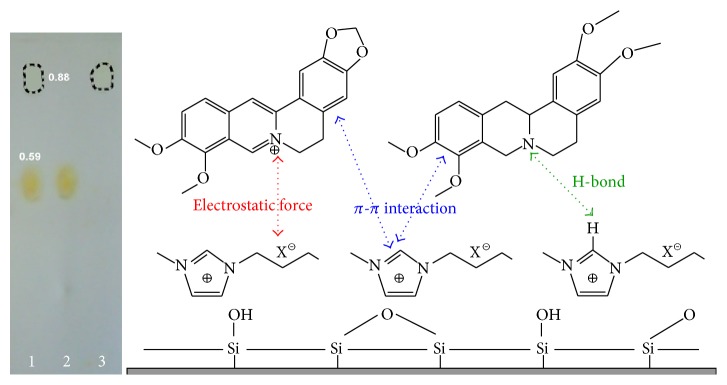
TLC chromatogram with [BMIM]OH : methanol (1 : 20, v/v) as developing reagent and separation mechanism (1: mixture of berberine hydrochloride and tetrahydropalmatine, 2: berberine hydrochloride, and 3: tetrahydropalmatine).

The application of ILs mobile phase could be easily realized on common silica thin layer plates, which was firstly tried to investigate separation performance for berberine hydrochloride and tetrahydropalmatine. Considering that the viscosity of pure ILs is relatively great, the developing agents (mobile phase) are selected to be the combination of ILs and popular alcohols. Methanol always has better solubility for ILs than ethanol and other higher alcohols, so various concentrations (1 : 20, 1 : 40, 1 : 60, 1 : 80, and 1 : 100, V/V) of [BMIM]OH, [BMIM]BF_4_, [BMIM]Br, and [BMIM]PF_6_ methanolic solutions were compared about the shape and *R*_*f*_ value of target spots together with the developing duration. As two traditional developing reagents,* n*-hexane-chloroform-methanol (10 : 6 : 1, V/V, for tetrahydropalmatine) and* n*-butanol-acetic acid-water (7 : 1 : 2, V/V, for berberine hydrochloride) [16, 17] were used for comparison. In the developing process, the major driving forces for berberine hydrochloride and tetrahydropalmatine could include electrostatic force and *π*-*π* interaction between them and the ILs (as shown in Figure 1), which have been discovered in similar researches [18, 19]. Because berberine hydrochloride has the structural character of quaternary ammonium, which makes it have greater polarity and stronger interaction with supported phase, its migration rate and *R*_*f*_ value (0.59) are lower than those of tetrahydropalmatine (*R*_*f*_ 0.88). Compared with conventional developing system, [BMIM]OH-methanol has simpler composition and is more suitable for simultaneous analysis of two target constituents in a plate. Without any extra pH additives, the shape of spots is ideal and no tailing occurs.

**Figure 2:**
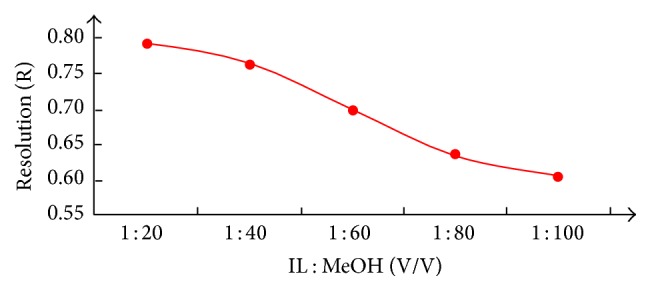
Effect of [BMIM]OH concentration on *R*_*f*_ values of berberine hydrochloride.

Moreover, [BMIM]OH is a kind of basic ionic liquid compared with the other three neutral ones. A part of its effects is supposed to be similar to the effect of amines as pH adjuster in those popular multicomponent developing reagents for berberine hydrochloride (like ammonia in the system of “benzene-ethyl acetate-methanol-isopropanol-strong ammonia (12 : 6 : 3 : 3 : 1, V/V)” or diethylamine in “ethyl acetate-chloroform-methanol-diethylamine (8 : 2 : 2 : 1, V/V)”) [17, 20]. Meanwhile, the amount of IL would obviously influence the migration distance of the alkaloids (see Figure 2). The result proves the proportional relationship of *R*_*f*_ value of berberine hydrochloride and [BMIM]OH concentration, and IL plays a more important key role in the developing system than methanol.

For the three neutral ILs, the resolution results indicated that the tailing of target spots could not be effectively inhibited through their interaction with Si-OH groups. In order to improve the shape of spots, 0.06 mol/L Na_2_HPO_4_-H_2_O buffer solution was added in methanol solution of these neutral ILs. As a result, the extent of improvement was in the following order: [BMIM]BF_4_ system was superior to [BMIM]Br system, which was superior to [BMIM]PF_6_ system. The performance of [BMIM]BF_4_-buffer salt system was relatively closest to that of [BMIM]OH system, but the developing time was greatly prolonged for the existence of water, which was double as much as that with [BMIM]OH-MeOH as developing reagent (40 min, developing distance = 10 cm). It is a very long time and unacceptable for TLC analysis. So [BMIM]OH was finally selected in the following study as stationary phase.

### 3.2. Effect of Reaction Time for the Preparation of SILs

**Figure 3:**
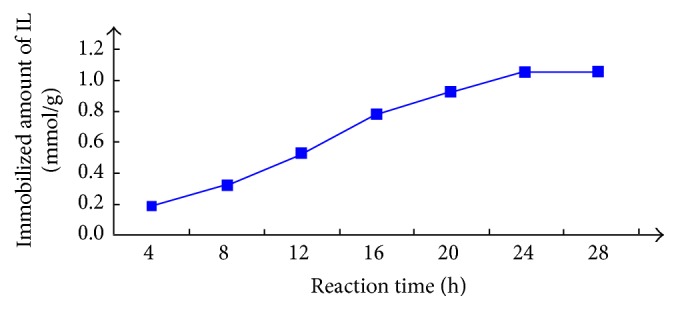
The relationship of immobilized amount and reaction time.

Through the forward description of the preparation of [BMIM]OH based SIL (SiO_2_·Im^+^·OH^−^), Im^+^·Cl^−^ was added to the silica gel suspension in anhydrous toluene and this mixture needed to be refluxed for a very long time for the immobilization of ionic liquid moieties onto silica gel. From 0 to 30 h, the products of SiO_2_·Im^+^·Cl^−^ were collected per 4 hours and each sample was analyzed with EA3000 elemental analyzer. The contents of ionic liquids attached to the silica surface were calculated on the basis of the percentage amounts of nitrogen (N%). The relationship of immobilized IL amount and reaction duration is shown in Figure 3. The increase of immobilized amount is not obvious after one day, so 24 h is selected finally. In the future study, the high-efficient catalyst and reaction condition (like microwave reactor) are expected to shorten such a long time.

### 3.3. Characterization of SILs

**Figure 4:**
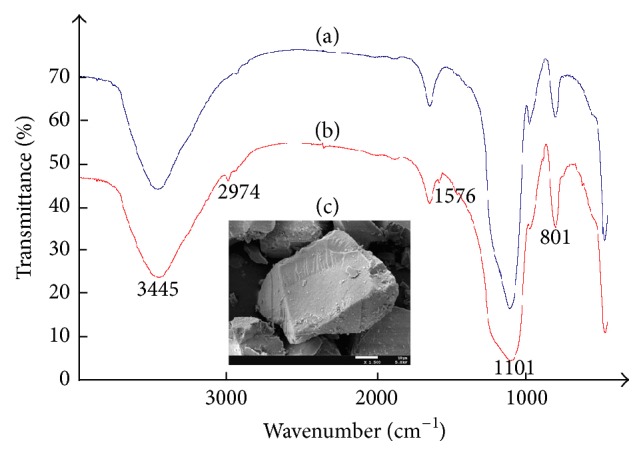
IR spectrum of blank silica gel (a) and SIL (b) together with SEM of SIL (c).

The elemental contents of SiO_2_·Im^+^·OH^−^ final product were analyzed, and the content of ionic liquid attached to the silica surface was obtained as 1.072 mmol/g. From the SEM photo (×1500, Figure 4), it can be seen that the surface of SiO_2_·Im^+^·OH^−^ particles is relatively smooth, which is different from the surface of common amorphous silica powders with many grooves and pores. A thermogravimetric study was performed to evaluate the thermal stability of SiO_2_·Im^+^·OH^−^, and its TGA curve only showed an initial loss of weight at temperature below 200°C, which was attributed to the removal of physically adsorbed water. Moreover, infrared spectrometry is one of the useful tools to identify the chemical modifications of silica gel. Small differences in wave numbers and intensities of the absorption bands are observed in the spectra of activated silica and immobilized ILs. In this study, FT-IR spectra of the activated silica and SILs were recorded between 400 cm^−1^ and 4000 cm^−1^, which were shown in Figures 4(a) and 4(b). In the spectrum of anchored [BMIM]OH surface, the finger-print region of the amide bands was from 1500 to 1600 cm^−1^; 1576 cm^−1^ is attributed to the characteristic frequency of the imidazolium groups, and 2974 cm^−1^ is assigned to the C-H stretching of the tetrahedral carbon. These IR peaks are similar to those reported in previous references [7, 21] and no obvious change of IR spectrum was observed in dry and sealed environment within 1 week. The above results can confirm the successful and stable anchoring of the IL onto the silica surface.

Other related parameters of SiO_2_·Im^+^·OH^−^ were listed in Table 1. On the basis of N_2_ adsorption-desorption isotherms (ASAP2020-M Micromeritics Co., Ltd., USA), pore size and pore volume were found and calculated by BJH (Barrett-Joyner-Halenda) method based on the desorption branch of the isotherms, and the specific surface area was calculated by Brunauer-Emmett-Teller (BET) model [22].

### 3.4. Separation and Quantitation of Alkaloids on IL Stationary Phase

There are two main immobilization modes of IL on TLC silica gel which have been studied, including physical coating and chemical bonding. In the former pattern, the selected ionic liquid was firstly mixed with small volume of 0.3% CMC-Na and then the mixture solution was added in plenty of 0.3% CMC-Na solution and fully stirred. Subsequently, the slurry was spread on glass flakes and the physical coated IL-SiO_2_ plates were prepared. However, this kind of TLC plates is found to be unstable and of low reproducibility according to our experiments. In the developing process, the IL layer on the surface of silica gel particles could easily fall off and be developed with those mobile phases containing excessive high-polar solvents, which would result in complex and uncontrollable separation mechanism. So the chemical bonding is recommended and selected in the following study.

**Figure 5:**
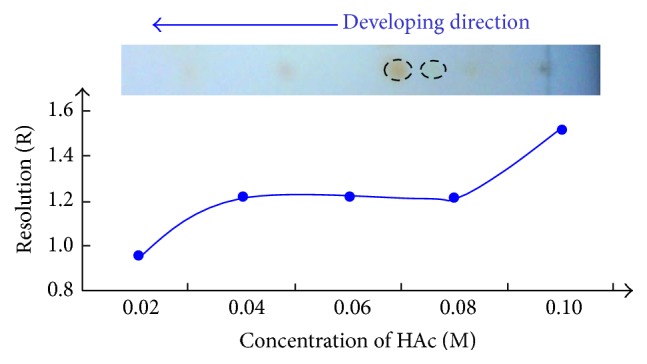
Effect of HAc concentration on resolution (L: berberine hydrochloride, R: tetrahydropalmatine, and developing distance = 8 cm).

The stationary phase of IL is of promise for having multimodal separation properties, which are seemingly more complex than that of IL mobile phase. In the developing process, the target compounds in “Stomacheasy” capsule were separated according to different levels of electrostatic force, hydrophobic interaction, and *π*-*π* interaction resulting from aromaticity of the rings in the structures of IL and alkaloids. These interactions provided by ILs have been reported in previous references [23, 24]. When SiO_2_·Im^+^·OH^−^ was used to separate the two alkaloids, the performance of different developing reagents was compared, which included the aqueous solutions of HAc, HCl, NaOH, and NaHCO_3_. It was found that HAc could give better separation trend among these candidate developing reagents. Moreover, the sequence of berberine hydrochloride and tetrahydropalmatine spots was inverted as can be expected, and the whole TLC separation system reflected a reverse-phase mechanism at some extent. The effect of HAc concentration for the resolution of berberine hydrochloride and tetrahydropalmatine was investigated as shown in Figure 5. Multiple interactions from SIL make the two alkaloids strongly retained, so they can not be developed and well separated when HAc concentration is low. When the concentration of HAc was above 0.1 M, the resolution of the two spots began to be greater than 1.5, which could meet the requirement of quantitative analysis. This level of HAc concentration can ensure complete ionization and the solubility difference between two ionized compounds in mobile phase reaches maximum. Berberine hydrochloride has greater polarity and solubility in polar developing system. So its *R*_*f*_ value is higher than that of tetrahydropalmatine. As a result, 0.1 M HAc aqueous solution was finally chosen as the developing reagent. It is worth mentioning that SiO_2_·Im^+^·OH^−^ plates also have better resolution between target alkaloids and other interference compounds in the capsules than ordinary TLC plates developed with those mentioned traditional developing reagents.

Under the above separation conditions, the contents of berberine hydrochloride and tetrahydropalmatine in the capsules were determined in the mode of zigzag scanning with single-wavelength reflecting method on CS-9301 flying spot scanner (light type: deuterium lamp, wavelength: 360 nm, beam size: 0.4 mm × 0.4 mm, SX = 7, and background subtraction). On the basis of prepared standard solutions, the standard curves of the two alkaloids were *y* = 2452.09*x* + 1979.28 for berberine hydrochloride and *y* = 1079.31*x* + 1288.23 for tetrahydropalmatine, respectively. As a result, the average contents were determined as 1.502 mg (berberine hydrochloride, 95% confidence interval, 1.494–1.510) and 0.747 mg (tetrahydropalmatine, 95% confidence interval, 0.741–0.753) per capsule according to their standard curves with external standard two-point method, which were close to the average values (*n* = 3) of 1.496 and 0.729 mg per capsule determined by the traditional TLC analysis in Section 2.5. According to the analysis results by statistical tools, 95% reliability could be obtained and a relatively larger *P* value (>0.05) indicated no obvious difference between the two methods. In a word, for Chinese patent medicine (CPM) with many coexisting constituents, SIL system had better resolution between target alkaloids and other interference compounds in the capsule than ordinary TLC plate developed with traditional developing reagents. Through the comparison of quantitative results on SIL plates and common silica plates, the new method together with its quantitative results on SIL plates was reliable.

### 3.5. Methodological Validation

The quantitative method on this kind of IL stationary phase showed a good correlation coefficient (*r* ^2^ = 0.9971~0.9976) in the concentration range 90~900 ng/spot with respect to peak area. Individual analyses of each sample were repeated in five replicates to evaluate their repeatability, and the peak areas %RSD of berberine hydrochloride and tetrahydropalmatine were 0.88% and 0.79%, respectively. The stability of peak areas was acceptable within eight hours. At three different concentration levels, measurement of peak area showed low values of standard error (SE) and %RSD (<1%) for variation of the analysis on the same and various plates. For these two target alkaloids, average of %RSD for intra- and interday precision of the quantitative method was determined to be 0.72% and 0.75%, respectively. Recovery of 95.91~104.85% (berberine hydrochloride) and 96.02~102.18% (tetrahydropalmatine) was obtained, which could indicate the method accuracy.

## 4. Conclusions

For the first time, [BMIM]OH and SiO_2_·Im^+^·OH^−^ were used in thin layer chromatography to analyze berberine hydrochloride and tetrahydropalmatine as mobile and stationary phase, respectively. The results of FT-IR, elemental analysis, and SEM confirm that the target ionic liquid has been successfully bonded onto the silica surface. The average contents of the two alkaloids in the Chinese patent medicine of “Stomacheasy” capsule were determined to be 1.502 mg/grain (berberine hydrochloride) and 0.747 mg/grain (tetrahydropalmatine) by TLC scanner on SiO_2_·Im^+^·OH^−^. The key conditions and chromatographic behaviors were also investigated in detail, and methodological validation proved the new TLC media was reliable and stable. Compared with traditional TLC analytical conditions, the simultaneous analysis with satisfied resolution can be achieved with simple developing reagent in two modes, which also can be easily prepared by common labs. This work is expected to provide useful reference for the application of IL and its bonded silica gel in related analytical fields.

## Figures and Tables

**Table 1:** Main related parameters of SiO_2_·Im^+^·OH^−^.

C (%)	H (%)	N (%)	Immobilized IL amount (mmol/g)	Specific surface area (m^2^/g)	Average pore size (nm)	Pore size (cm^3^/g)	Ash content (%, 800°C)
10.334	2.827	3.168	1.072	198	5.342	0.618	82.5
